# Targeted and immunotherapeutic strategies for castration-resistant prostate cancer: emerging strategies, challenges, and future directions

**DOI:** 10.3389/fimmu.2025.1668188

**Published:** 2025-10-22

**Authors:** Bo Wang, Yuchu Xiang, Zitong Fang, Junjun Le, Yu Jian, ShuLian Chen, Daobing Li, Guobiao Liang, Xiaoting Pan

**Affiliations:** ^1^ Department of Urology, Affiliated Hospital of Zunyi Medical University, Zunyi, Guizhou, China; ^2^ Institute of Medical Microbiology and Hygiene, Faculty of Medicine, University of Freiburg, Freiburg, Germany; ^3^ Department of Dermatology, West China Hospital, Sichuan University, Chengdu, China; ^4^ Laboratoryof Dermatology, Clinical Institute of Inflammation and Immunology, Frontiers Science Center for Disease-related Molecular Network, West China Hospital, Sichuan University, Chengdu, China; ^5^ College of Life Sciences, Sichuan University, Chengdu, China; ^6^ Department of Clinical Medicine, Shanghai Medical College, Fudan University, Shanghai, China

**Keywords:** castration-resistant prostate cancer, immunotherapy, targetedtherapy, emerging strategies, challenges, future directions

## Abstract

Castration-resistant prostate cancer (CRPC) represents an advanced stage of prostate cancer progression. Although the combination of androgen deprivation therapy (ADT) with chemotherapy and first generation hormone therapy is initially effective, patients ultimately develop resistance. In recent years, breakthroughs in targeted therapies and immunotherapies, along with the emergence of novel combination strategies, have provided new hope for patients with CRPC. This article systematically reviews the latest advancements in targeted and immunotherapeutic approaches for CRPC, integrating clinical data and mechanistic studies to analyze the efficacy and challenges of novel agents (e.g., second-generation AR inhibitors, PARP inhibitors, PSMA-targeted therapies) and combination regimens. It also provides insights for exploring future optimization directions.

## 1 Introduction

As of 2020, prostate cancer is the most frequently diagnosed cancer among men in 112 countries. It represents one in every 14 cancers diagnosed worldwide and accounts for 15% of all cancers in men. Androgen deprivation therapy (ADT) is an important treatment for prostate cancer by reducing the levels of androgens in the body to inhibit the tumor growth. The disease can be categorized based on its response to ADT: Castration-Sensitive Prostate Cancer (CSPC) and Castration-Resistant Prostate Cancer (CRPC). CSPC is an early stage of metastatic prostate cancer, in which ADT, as an important treatment method for cancer, can effectively inhibit tumor growth by reducing testosterone levels in the body. Concurrently, chemotherapy and next-generation hormone therapy have been extensively utilized at this stage, as the efficacy of these treatments continues to be validated (1). Castration-resistant prostate cancer (CRPC), as an advanced stage of prostate cancer progression, is recognized as: castration level testosterone ≤50 ng/dL (or ≤0.50 ng/mL or 1.73 nmol/L) with prostate specific antigen (PSA) progression of at least a 25% increase in PSA from nadir (st ting PSA ≥1.0 ng/ml) or radiological progression (2). Genetic instability, chromosomal aberration, remodeling of the tumor microenvironment, alterations in androgen receptor (AR) signaling, dysregulation of additional genes and DNA damage response (DDR) are considered to constitute a complex pathogenesis of CRPC (3). Chemotherapy based on taxane regimens and endocrine therapy, which have been continued and refined from CSPC stage, are widely used in the therapy of CRPC, but the tumor response rate (RR) and overall survival (OS) for patients are limited (4). In recent years, the breakthrough of new drugs in targeted therapy and immunotherapy, as well as the emergence of new drug treatment strategies have provided new hope for patients with CRPC. By integrating the latest clinical data and mechanistic studies, we analyze the efficacy and challenges of novel agents and combination therapeutic strategies. Additionally, we explore future optimization directions to provide insights for clinical practice and scientific research.

## 2 Targeted therapy in CRPC

**Figure 1:**
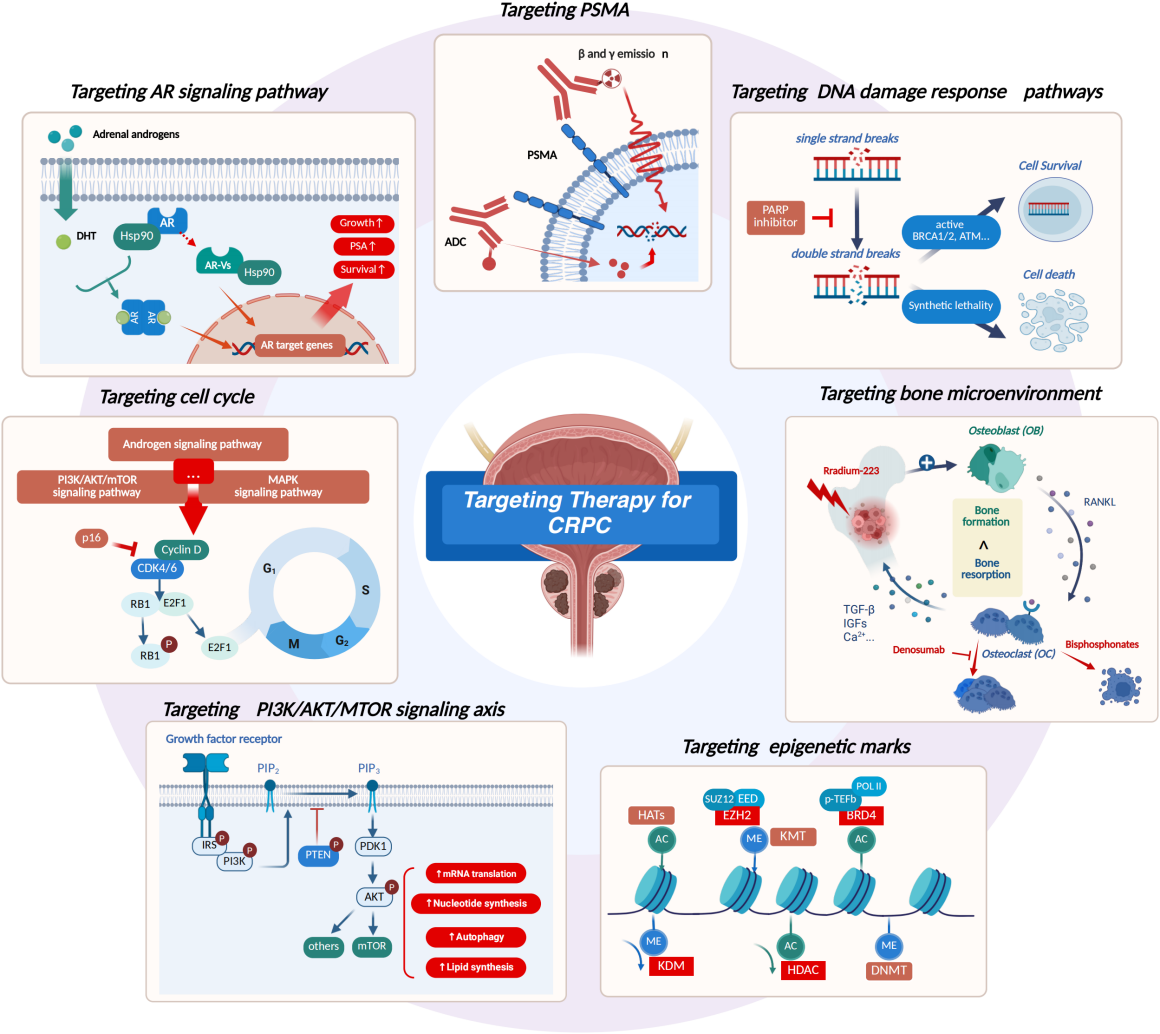
Overview of the targeted therapies for CRPC. (1) Targeting AR signaling pathway: inhibition of DHT production, inhibition of AR amplification, inhibition of AR action on genes, and degradation of AR variants. (2) Targeting DNA damage response pathways: inhibit poly ADP-ribose polymerase to cause synthetic lethality. (3) Targeting PSMA: Identify PSMA (Prostate-specific membrane antigen) to introduce beta and gamma emissions and ADC drugs. (4) Targeting bone microenvironment: targeting RANKL, regulate the proliferation and death of osteoclasts, or introducing radiation directly targeting bone tumors. (5) Targeting cell cycle: inhibite the transition from the G1 phase (gap 1) of cell cycle to S phase (DNA synthesis). (6) Targeting PI3K/AKT/MTOR signaling axis: inhibit PI3K (Phosphatidylinositol 3-kinase), AKT (as same as Protein Kinase B, PKB) and mTOR (mammalian target of rapamycin), especially when PTEN (Phosphatase and tensin homolog) deletion. (7) Targeting epigenetic marks: Inhibiting EZH2 (Enhancer of zeste homolog 2) to clear methylation, inhibiting BRD4 (Bromodomain-containing protein 4) to clear acetylation, KDM (histone lysine methyltransferase) promotes demethylation, and HDACs (Histone Deacetylase) can clear histone acetylation. CRPC, castration-resistant prostate cancer. Created in https://BioRender.com.

Targeted therapies work by specifically blocking key molecular pathways that drive tumor growth and metastatic, including androgen receptor-targeted therapy, DNA damage response-targeted therapy, prostate-specific membrane antigen-targeted therapy, bone microenvironment-targeted therapy. In addition, as biomedical research and therapeutic development continue to advance, another three key strategies have emerged as new areas of focus: cell cycle modulation, PI3K/AKT/mTOR pathway targeting, and epigenetic marker regulation (**Figure 1**). Many targeted therapeutic drugs are gradually being used not only in CRPC, but also CSPC.

### 2.1 Targeting AR signaling pathway

The androgen receptor (AR) is a ligand-dependent transcription factor and belongs to the family of steroid receptors, which is consisted of DNA-binding domain (DBD), carboxy-terminal ligand-binding domain (LBD), NTD (N-terminal domain) and hinge region of the AR In prostate cancer, the LBD is the most frequent site of gain-of function mutations. The LBD is the place that interacts with testosterone and dihydrotestosterone (DHT), and also with a number of drugs including bicalutamide, flutamide, and nilutamide by competitively inhibiting the binding site of transcription-activating androgens (5).While the carboxyl terminus and DBD structure are already clear, the unknown amino terminus hinders the development of amino terminus targeted drugs (6).Under physiological conditions, the principal androgenic ligands for AR are testosterone and its more potent 5a-reduced derivative, DHT, androgen binding triggers conformational changes that promote AR nuclear translocation, homodimerization, binding to DNA at androgen response elements, and direct transcriptional activation of target genes (7).Under pathological conditions, AR can affects the development of prostate cancer by regulating transcriptional networks, genome stability, and DNA repair, as evidenced by the emergence of gene fusion. AR can affects the development of prostate cancer by regulating transcriptional networks, genome stability, and DNA repair, as evidenced by the emergence of gene fusion (8). In 1941, Huggins and Hodges have demonstrated for the first time that ADT can effectively treat prostate cancer. ADT can suppress serum testosterone to castration levels and thus block the activation of the AR.

But in CRPC, amplification, LBD mutations, constitutively active AR variants (AR-Vs) and alterations in pathways of androgen biosynthesis of AR and so forth can lead to the AR pathway alterations and increased AR signaling, and results in the failure of the original ADT treatment (7). Treatment-associated changes converged upon the AR gene, dominant AR genotype continues to evolve during sequential lines of AR inhibition and drives acquired resistance in patients with CRPC (9). ADT-induced AR gene transcription rate and recruitment of splicing factors to AR pre-mRNA contribute to enhancing AR-V7 levels in prostate cancer cells, thereby leading to the development of drug resistance (10).But CRPC-like cells are reported that it present early in the development of PCa and are not exclusively the result of acquired evolutionary selection during ADT, which may require aggressive early intervention (11). To address the changes in AR axis in CRPC, more potent AR antagonists and inhibitors have been developed to block the AR axis and inhibit tumor growth.

1) Abiraterone acetate (ABI) is a specific inhibitor of cytochrome P (CYP) 17, a critical enzyme in androgen synthesis (4). Abiraterone acetate prolongs the overall survival of CRPC patients who have previously received chemotherapy by inhibiting the synthesis of androgens, a viewpoint that has been validated in the early 2010s (12). A 2022 article suggests that in high-risk non metastatic prostate cancer patients, Abiraterone acetate with prednisolone is associated with significantly higher metastasis free survival rates compared to ADT alone treatment. This demonstrates that AbIIalso has the ability in CSPC (13). However, accelerated abiraterone metabolism results in a decline of plasma abiraterone as disease progresses. This may lead to the development of drug resistance (14).

There are other CYP17A1 inhibitors have also been developed, such as orteronel (TAK-700) and galeterone (TOK-001). In the phase III study, Orteronel showed longer radiographic progression-free survival (rPFS) and higher PSA50 rate after combined treatment with prednisone, indicating its anti-tumor activity (15).The efficacy and safety from ARMOR1 and ARMOR2 part 1 and the pharmacokinetic results support the galeterone tablet dose of 2,550 mg/d for further study (16).Galeterone did not demonstrate stronger therapeutic efficacy than Enz in AR-V7-positional (AR-V7+) Castration-Resistant Prostate Cancer (mCRPC) (17).But a phase III study targeting the entire mCRPC population has not yet been conducted.

2) Enzalutamide (ENZ), a second-generation AR inhibitor, exerts its therapeutic effects by targeting multiple critical steps in the AR signaling pathway. Specifically, by binding to the LBD of AR, it inhibits the binding of androgens to the AR, prevents the nuclear translocation of the activated AR, and disrupts the binding of the activated AR to its target DNA sequences. It is more effective compared to first-generation AR antagonists like bicalutamide, nilutamide and flutamide (18). Since 2013, the effectiveness of ENZ in the treatment of CRPC has been widely confirmed (19). ENZ treatment maintains lower levels of pain and burden of prostate cancer symptoms, as well as higher health-related quality of life (20). And in the PREVAIL study with >5 yr of follow-up, ENZ continued to demonstrate improved survival in patients with mCRPC. However, ENZ was associated with an increased rate of fatal adverse events during treatment, particularly fatal cardiovascular events (1.6% vs 0.4%) (21). The beneficial effect of enzalutamide on mCSPC patients has also been confirmed, and it is not related to the HSD3B1 genotype, although HSD3B1 may be associated with lower response rates to abi or ENZ therapy for CRPC (22, 23). When ABI and ENZ were used in combination, a longer time to second PSA progression for the sequence of ABI followed by ENZ than with the opposite treatment sequence. Using a sequencing strategy of ABI followed by ENZ provides the greatest clinical benefit (24, 25). The simple combination of ENZ, ABI, and prednisone does not prolong overall survival (OS) and may lead to higher non hematological toxicity (26).

3) Apalutamide (APA) is a next-generation AR inhibitor with higher affinity to the AR. It is a competitive AR inhibitor that is fully antagonistic to AR overexpression, a common and important feature of CRPC (27). A multicenter, open-label, phase Ib drug-drug interaction study discovered with 1,000 mg ABI plus 10 mg prednisone daily with 240 mg APA daily was well tolerated and showed evidence of antitumor activity in patients with mCRPC, including those with disease progression on AR signaling inhibitors. And APA decreased exposure to prednisone (28, 29). In the placebo-controlled SPARTAN study, apalutamide plus ADT did not increase detectable AR/non-AR aberrations over ADT alone (30).

4) Darolutamide (DAR) is a more potent AR antagonist than ENZ or APA which can antagonizes mutated AR. In the planned primary analysis of a phase 3 trial, overall survival at 3 years was 83% (95% confidence interval in the DAR plus ADT group and 77% in the placebo plus ADT group. And there was no significant difference in the incidence of adverse events (31). In men with mCRPC, DAR was associated with a clinically meaningful benefit in episodic memory and less fatigue compared with ENZ (32). DAR inhibited the transcriptional activity of AR mutants in the plasma of CRPC patients receiving traditional treatment. In particular, DAR significantly inhibited the transcriptional activity of F877L, H875Y/T878A, F877L/T878A, and the previously unreported T878G AR mutant, which converted ENZ into a partial agonist (33).

ABI as well as ENZ are the typical second-generation AR-targeted agents, which continuously show their effectiveness in the therapy of CRPC. But the innate resistance or acquired resistance are still a serious therapeutic challenge. Novel strategies for prostate cancer therapy are required to overcome resistance to next-generation AR inhibitor. For example:

5) AR-Vs isoforms of the AR lacking a LBD and remain constitutively active in the absence of circulating androgens, thus promoting cancer cell proliferation, that is not inhibited by anti-androgen therapies, including abiraterone or enzalutamide. The mutation of splicing regulatory factors, changes in splicing regulatory factor activity, and alternative splicing of cell signaling pathways have led to the progress of CRPC. Bavdegalutamide (ARV-110), is a proteolysis targeting Chimera (PROTAC^®^) protein degrader that recruits the cereblon-containing E3 ubiquitin ligase to direct the polyubiquitination and subsequent proteasomal degradation of AR. Promising preclinical data supports the clinical development of bavdegalutamide as a potential therapeutic drug for prostate cancer patients (34). Related clinical studies are currently underway (NCT05177042, NCT03888612). An ultrasmall gold (Au)-peptide complex platform to deliver the peptide-based proteolysis-targeting chimera (PROTAC) *in vivo* is developed and it is designed to induce AR and AR-V7 degradation in a DBD and MDM2-dependent manner, without showing any activity on other hormone receptors (35). A novel cereblon-based AR degrader, UBX-390, is presented as an optimized AR degrader with remarkable potential for treating CRCP (36). Rutaecarpine, as one of the main components of Evodia rutaecarpa, selectively induces AR-V7 protein degradation through K48 linked ubiquitination, and also has certain clinical application prospects (37).

### 2.2 Targeting DNA damage response pathways

The sustained attack of endogenous and exogenous damage to the genome can lead to single strand breaks (SSB) and double strand breaks (DSB), and DNA damage response (DDR) is a complex biochemical pathway system evolved by cells to respond to such attacks (38). However, genomic instability caused by DDR defects is a hallmark of cancer, with a higher mutation burden increasing the chances of oncogene activation and loss of tumor suppressor genes, leading to tumorigenesis. The genetic diversity of cancer cell populations within tumors also increases the chance of developing drug-resistant clones after radiotherapy or chemotherapy, thereby promoting cancer recurrence (39).Some studies shown that approximately 23% of metastatic castration resistant prostate tumors harbor loss­of­function somatic or germline alterations in DDR genes such as BRCA2, BRCA1, ATM, HOXB13 and CHEK2.3 (40).Among them, the mutations that confer the highest risk are those in BRCA2 and HOXB13, which confer a sevenfold to eightfold and threefold increased relative risk, respectively (41).This provides new ideas for drug development for our clinical work.

Poly ADP-ribose polymerase (PARP), a protein that is important for repairing DNA single-strand breaks, resulting in synthetic lethality (42).The CRPC patients with DDR gene aberrations can be therapeutically used with PARP inhibitors to induce synthetic lethality. Multiple PARP inhibitors including Olaparib, Rucaparib, Talazoparib, Niraparib and Pamiparib have demonstrated potential applications in clinical trials.

In TOPARP-B studies, the clinical benefit of Olaparib monotherapy in mCRPC has been unequivocally shown for patients with DNA repair defects (43).Most benefit was seen among patients with BRCA2 homozygous deletions, biallelic loss of PALB2, and loss of ATM protein. In addition, loss of RAD51 foci, evaluating homologous recombination repair function, was found primarily in tumors with biallelic BRCA1/2 and PALB2 alterations (44).Similar conclusions also appear in PROfound that among men with metastatic castration-resistant prostate cancer who had tumors with at least one alteration in BRCA1, BRCA2, or ATM, those who were initially assigned to receive olaparib had a significantly longer duration of overall survival than those who were assigned to receive enzalutamide or abiraterone plus prednisone as the control therapy (45).Olaparib was associated with reduced pain burden and better-preserved HRQOL compared with the ABI and ENZ (46). The TRITON-2 phase 2 trial evaluated rucaparib 600 mg twice daily in patients with mCRPC, which shows the 43.5% of objective response rate (ORR) (95%CI 38.1- 63.4%) for the 62 ORR-evaluable patients with a BRCA1/2 alteration, and 4.1%, 6.7% and 16.7% in the ATM group, CDK12 group and CHEK2 group, respectively. The effector genes of the HRR system (BRCA, PALB2) appear to have higher efficacy in CRR than sensors (ATM, CDK12) (47). A meta-analysis including 17 clinical trials showed that PARPi monotherapy improved rPFS and OS in mCRPC patients with alterations in BRCA1 or BRCA2 genes but not in those with alterations in the ATM gene (48).The similar efficacy of Niraparib, Talazoparib have also been confirmed in GALAHAD studies and TALAPRO studies (49).

The combined use of PARPIs with ENz and ABI has also been recognized for its benefits. The TALAPRO-2 trial was a randomized, placebo, controlled, multicentric, multinational, double-blind, phase 3 trial which proved that Talazoparib + Enza combination gives promising result with a significant improvement in rPFS in comparison to enzalutamide monotherapy as first-line treatment in mCRPC. This combination lowered the risk of radiographic progression or death by 37% with minimal side effects, especially in mCRPC patients carrying HRR gene alterations (50–52). In PROpel, Olaparib plus abiraterone resulted in a statistically significant and clinically meaningful improvement in radiographic progression-free survival versus active standard-of-care abiraterone as first-line treatment for mCRPC, but Overall survival was not significantly different between treatment groups at this final prespecified analysis (53). A meta-analysis including 17 clinical trials showed that PARP inhibitors increased the risk of myelodysplastic syndrome and acute myeloid leukemia versus placebo treatment (54). Anemia, neutropenia, and fatigue are the common adverse drug events (ADEs) of PARP inhibitors (52). Other related adverse effects should be further studied. Given that PARP inhibitors have not shown significant improvement in hard outcomes such as overall survival or quality of life, caution should be exercised when using these treatment methods in routine clinical practice for patients without BRCA1/2 mutations (55).

PARPIs has also made progress in the research of combination therapy. Cediranib, a pan-vascular endothelial growth factor receptor inhibitor. Cediranib combined with olaparib improved rPFS compared with olaparib alone in men with mCRPC. This combination was associated with an increased incidence of grades 3–4 adverse events (56).Higher rates of PSA50 and ORR were reported in participants treated with PARPi + androgen receptor signaling inhibitor (ARSI) than in single-agent PARPi or PARPi + immune checkpoint inhibitors (ICIs) (48). Combining niraparib with Radium-223 in patients with mCRPC was safe. Whole blood gene expression of PAX5 and CD19 was higher in responders and ARG-1, IL2R, and FLT3 expression was higher in nonresponses. However, further studies incorporating biomarkers will better elucidate the role of combinations of PARP inhibitors with DNA damaging and other agents (57). CheckMate 9KD (NCT03338790) is a nonrandomized, multicohort, phase 2 trial of nivolumab plus other anticancer treatments for metastatic castration-resistant prostate cancer (mCRPC). Nivolumab plus rucaparib is active in patients with HRD-positive post-chemotherapy or chemotherapy-naive mCRPC, particularly those harboring BRCA1/2 mutations. But whether the addition of nivolumab incrementally improves outcomes versus rucaparib alone cannot be determined from this trial (58).

However, PARP inhibitors still develop resistance during use BRCA reversal mutation is a known mechanism of acquired resistance to PARP inhibitors in various cancer types, which has also been confirmed in CRPC (59).The occurrence of BRCA reversal mutations and their relationship with clinical benefits still require further research, which may become a key factor in developing effective therapies for drug-resistant diseases. Loss of CHEK2 confers resistance rather than sensitivity to PARP inhibition through increased expression of BRCA2, a target of CHEK2-TP53-E2F7-mediated transcriptional repression. Combined PARP and ATR inhibition overcomes PARP inhibitor resistance caused by CHEK2 loss (60).

In addition, targeting the DDR in cancer is not confined to PARP inhibition, as other potential DDR targets have been identified in recent years; small-molecule inhibitors of several targets have been developed, and some are being tested in clinical trials (39). Pharmacologic inhibition of DNA-dependent protein kinase catalytic (DNA-PKc), a component of Non-homologous end joining (NHEJ) repair machinery, with all three inhibitors, significantly resensitized DU145-DxR (prostate cancer cell line model of docetaxel-resistance) to taxane (61).The efficacy of M3814 which is a DNA-PKc inhibition, is validating in NCT04071236. In another Phase 1b multicentre trial evaluating enzalutamide with escalating doses of CC-115 which is a dual mTORC1/2 and DNA-PK inhibitor in AR inhibitor-naive mCRPC patients.

### 2.3 Targeting PSMA

Prostate-specific membrane antigen (PSMA), also known as glutamate carboxypeptidase II (GCPII) or N-acetyl-L-aspar- tyl-L-glutamate peptidase I (NAALADase I), is a 750 amino-acid type II transmembrane glycoprotein, located in three domains, including the intracellular domain that contains 19 amino acids, the transmembrane domain that consists of 24 amino acids, and the extracellular domain containing 707 amino acids (62).It can be found in normal prostatic tissue and the vascular endothelium in a wide variety of solid tumors, but not in blood vessels of normal tissues (63). PSMA is expressed at a very low level in normal prostatic tissues and non-prostatic tissues, but its expression in prostate cancer (Pca) tissues increases by 100–1000 times compared to that in normal tissues (64).This distribution characteristic has attracted attention to tumor therapy targeting PSMA. There are three main types of ligands that can be used to target PSMA: monoclonal antibodies, aptamers, and small molecule inhibitors. monoclonal antibodies J591, Small molecule inhibitors such as glutamate-urea-lysine (Glu-urea-Lys), PSMA-I&T, PSMAI&S, and PSMA-617 have been widely used in targeted therapy of PCa (62).

Radioligand therapy (RLT) involves administering a therapeutic dose of a radionuclide-labeled ligand into the body. Once the ligand specifically binds to the target cells, the radionuclide emits alpha (α) particles, beta (β) particles, or Auger electrons. These emissions interact with biological macromolecules, generating free radicals that cause DNA single- or double-strand breaks. This process ultimately leads to aging, apoptosis, or necrosis of the targeted cells. ^177^Lu-PSMA-RLT is a radiolabeled small molecule that delivers β radiation to cells expressing PSMA. An open-label randomised phase 2 trial shown that ^177^Lu-PSMA-617 compared with cabazitaxel (a semi synthetic taxane with poor affinity for P-gp) in men with metastatic castration-resistant prostate cancer led to a higher PSA response and fewer grade 3 or 4 adverse events (65). But overall survival differed between the randomised groups did not be founded due to the small sample size and a median follow-up of 2.5 years (66). The present data indicate that ^177^Lu-PSMA-617 beneficial effects on OS are strongly influenced by pretreatment (history of second line chemotherapy with cabazitaxel) and the presence of visceral metastases at onset of ^177^Lu-PSMA-617 treatment (67). A nomogram to predict outcomes after Lu-177-PSMA in patients with mCRPC has been developed. And predictors included in the nomograms were time since initial diagnosis of prostate cancer, chemotherapy status, baseline hemoglobin concentration, and [Ga-68] Ga-PSMA-11 PET/CT parameters (molecular imaging TNM classification and tumour burden). These externally validated nomograms might help in clinical trial design and individual clinical decision making (68). In addition, besides 177Lu-PSMA-617, 225Ac-J591 and 177Lu-J591 have been studied in clinical trials (69). 177Lu-PSMA-617 has also been observed to play a role in metastatic hormone sensitive prostate cancer in experiments (70).

Furthermore, PSMA antibody-drug conjugate (PSMA ADC) is a fully human immunoglobulin G1 anti-PSMA monoclonal antibody conjugated to monomethylauristatin E, which binds to PSMA-positive cells and induces cytotoxicity. A phase 2 trial in metastatic castration-resistant prostate cancer (mCRPC) subjects who progressed following abiraterone/enzalutamide (ABI/ENZ) therapy confirmed that PSMA-ADC demonstrated some activity with respect to PSA declines, CTC conversions/reductions, and radiologic assessments in ABI/ENZ treated mCRPC subjects (71).

### 2.4 Targeting bone microenvironment

Over 90% of patients with metastatic castration-resistant prostate cancer (mCRPC) develop bone metastases, which impaired the structural integrity of the bone and often resulted in skeletal events associated with increased pain, poor quality of life (QOL), and reduced survival (72). Crucial determinants of bone health and disease, and specific alterations have been discovered in bone metastases, including activation of osteoclastic bone resorption, suppression of osteoblastic bone formation in osteolytic lesions, neo angiogenesis and aberrant osteoimmune interactions. The development of drugs targeting these mechanisms provides new and effective treatment strategies for prostate cancer bone metastasis.

1. Radium-223 dichloride (radium-223), a bone-targeted alpha-particle therapy. Alpha particles have a relatively shorter range, spanning 2–10 cell diameters with a higher linear energy transfer, thereby delivering a highly targeted effect with limited hematologic toxicity (73).The emitted high-energy alpha particles induce DNA double-strand breaks that might be irreparable and lead to cell death in nearby exposed tumour cells, osteoblasts and osteoclasts (74). Radium-223 high-dose or extended-schedule regimens resulted in no change in SSE-FS or other efficacy end points and were associated with more grade >= 3 TEAEs. Thus, the approved radium-223 regimen is 55 kBq/kg every 4 weeks (q4w) for six cycles (standard dose) according to the pivotal phase III ALSYMPCA trial (75, 76).Although the frequency of thrombocytopenia increased, treatment with radium-223 was well-tolerated without increasing the risk of leukemia or other cancers (77, 78).To personalize Ra-223 treatment, researchers confirmed that dosimetry may be useful to identify a more appropriate Ra-223 administered activity predicting adsorbed dose to target tissue and a dose dependent complex chromosome damage occurs during Ra-223 administration and this injury is more evident in heavily pre-treated patients (79).In addition, the efficacy and safety of Ra in combination with other drugs are currently under multiple explorations. To investigate the combination therapy of radium-223 and sipuleucel-T, in an open-label, phase II multicenter trial containing 32 people, Patients in the combination arm were more likely to have a >50% PSA decline(31% vs. 0), and also demonstrated longer Progression-Free-Survival (PFS) and OS, despite the paradoxically lower immune responses observed. Additional study to confirm these findings in a larger trial is warranted (80). Atezolizumab + radium-223, regardless of administration schedule, had greater toxicity than either drug alone, with no clear evidence of additional clinical benefit for patients with mCRPC and bone and lymph node and/or visceral metastases (81).
2. Bisphosphonates is a commonly used drug that inhibits osteoclast activity. The anti-tumour effects of bisphosphonates are multiple that besides impairing osteoclast mobility and adhesion, amino bisphosphonates can have direct effects on tumour cells and might have immunomodulatory effects, in particular, on macrophages and γδT cells (82). The third-generation bisphosphonate zoledronic acid was approved by the FDA to prevent skeletal-related events (SREs) in patients with mCRPC in 2002. 4 mg zoledronic acid can reduce the incidence of skeletal-related events in prostate cancer patients with bone metastasis (83).In a randomized, open-label clinical trial conducted at 269 academic and community sites in the United States, the use of zoledronic acid every 12 weeks compared with the standard dosing interval of every 4 weeks did not result in an increased risk of skeletal events over 2 years. This longer interval may be an acceptable treatment option (84).
3. Denosumab is a specific RANKL antibody denosumab that neutralizes the activity of RANKL. RANKL plays important role in bone metastases. RANKL/RANK signaling induces preosteoclast differentiation and maintains the survival and function of osteoclasts (82). The use of denosumab has been recommended in the 2011 EAU guidelines (85).Denosumab significantly delayed time to first bone metastasis, increased bone-metastasis-free survival by a median of 4·2 months compared with placebo, although the overall survival did not differ between groups (86). Minodronate or denosumab can be used not only to prevent and treat bone metastasis, but also to prevent ADT related bone loss in Asian PCa patients (87).Because denosumab treatment is associated with life-threatening hypocalcemia, proactive treatment of calcium and calcitriol should be considered when using denosumab (88).

In summary, considering skeletal‐related events, zoledronic acid and denosumab appeared to be the most effective, but also seemed to cause the most and worst adverse events (like renal impairment for treatment with zoledronic acid and osteonecrosis of the jaw for denosumab) (89). Therefore, benefits and risks should be evaluated in treatment selection.

### 2.5 Targeting cell cycle

Cyclin-dependent kinase 4 and 6 (CDK4/6) inhibitors radically changed the treatment paradigm for breast cancer. The androgen receptor of prostate cancer is similar to the estrogen receptor in breast cancer, which may activate the cyclin D-CDK4/6, driving the proliferation of prostate cancer and resistance to hormone manipulation (90).Therefore, this has stimulated the search for CDK4/6 inhibitors. Aberrant cellular proliferation, resulting from dysregulation of the processes controlling cell division, is one of the hallmarks of cancer. CDK4 and CDK6, two serine/threonine kinases, are crucial for governing the transition from the G1 phase (gap 1) of cell cycle to S phase (DNA synthesis) (91). In addition to the various mechanisms that control the expression, nuclear export, and degradation of D-type cyclins, CDK4/6 activity is also regulated by the INK4 (INK4B (p15), INK4A (p16), INK4C (p18) and INK4D (p19)) and WAF1 and KIP (p21 (WAF1), p27 (KIP1) and p57 (KIP2)) cyclin-dependent kinase inhibitor protein families (92). Complex interaction mechanisms all have the potential to be targets of CDK4 or CDK6 regulation.

A signal finding study demonstrated good tolerability of Abemaciclib monotherapy and demonstrated clinical activity. This study is considered preliminary proof-of-concept and designates CDK4/6 as a valid therapeutic target in prostate cancer (90). Another CDK4/6 inhibitor, Ribociclib, has been shown to have good efficacy and acceptable adverse reactions in mCRPC patients undergoing chemotherapy naïve and progression to ≥ 1 ARSI treatment with intermittent Ribociclib combined with docetaxel once every 3 weeks. Further evaluation is needed in randomized clinical trials (93).Clinical trials are underway for the synergistic treatment of Abemaciclib and Atezolizumab (NCT04751929), Abemaciclib and 177Lu PSMA-617(NCT05113537), and Abemaciclib and Abiraterone Acetate (NCT03706365). We look forward to the development of CDK4/6 inhibitors creating a new paradigm for the treatment of mCRPC.

### 2.6 Targeting PI3K/AKT/MTOR signaling axis

The PI3K/AKT/mTOR (PAM) signaling pathway is a highly conserved signal transduction network in eukaryotic cells that promotes cell survival, cell growth, and cell cycle progression. Dysfunction of components of this pathway such as hyperactivity of Phosphatidylinositol 3-kinase (PI3K), loss of function of Phosphatase and tensin homolog (PTEN), and gain-of-function of Phosphatidylinositol 3-kinase (AKT), are notorious drivers of treatment resistance and disease progression in cancer (94). PTEN, a dual specificity phosphatase, can act as a direct antagonist of class I PI3K activity, which converts PIP2 to PIP3. PTEN deficiency leads to abnormal accumulation of PIP3 on the cell membrane, causing PDK1 to recruit and phosphorylate its substrate AKT, which further activates mammalian target of rapamycin (mTOR) (95). PTEN deletion (40.7%) is a common recurrent somatic gene alteration in mCRPC amplification or activating mutations of PIK3CA, PIK3CB, PIK3R1 and AKT1are less common, being observed in <15% of patients (40). Dealing with changes in this pathway may bring new hope to patients with mCRPC.

Ipatasertib is a sort of AKT inhibitors which plus ABI significantly improved radiographical progression-free survival compared with placebo plus ABI among patients with mCRPC with PTEN-loss tumors, although there was no significant difference between the groups in the intention-to-treat population (96).Capivasertib is a pan-AKT inhibitor. ProCAIDs study indicate that Capivasertib to chemotherapy did not extend cPFS in mCRPC, but did significantly improve the secondary endpoint of OS (97, 98).In a trial of using samotolisib (PI3K/mTOR dual kinase and DNA-dependent protein kinase inhibitor) combined with ENZ to treat mCRPC patients who experienced cancer progression after treatment with ABI, the combination therapy was tolerable for adverse reactions, significantly improved PFS, and may occur in patients with intact PTEN and no androgen receptor splicing variant 7. Significant in patients without androgen receptor splicing variant 7 and with intact PTEN (99). The effectiveness of another pan-PI3K/mTOR inhibitor Gedatolisib in breast cancer was proved in Phase I study (100). And it’s trial in mCRPC is recruiting patients (NCT06190899). In addition, phase I trials of other drugs such as GSK2636771 and CC-115 have confirmed good tolerability, but further research is needed to determine their therapeutic effectiveness (101, 102).

### 2.7 Targeting epigenetic marks

Epigenetic controls of transcriptional regulation include DNA methylation, histone modification, and chromatin remodeling. These epigenetic modifications drive carcinogenesis in prostate cancers. Many epigenetic regulators and chromatin remodelers are mutated in up to 20% of advanced prostate cancers (103). Epigenetic reprogramming may mediate the transition of neuroendocrine prostate cancer (NEPC) and play a role in maintaining this treatment resistance state (104).

Enhancer of Zeste homolog 2 (EZH2) is a histone methyltransferase and emerging therapeutic target. EZH2 modulates bivalent genes that results in upregulation of NEPC-associated transcriptional drivers (e.g. ASCL1) and neuronal gene programs in NEPC, and leads to forward differentiation after targeting EZH2 in NEPC (105). EZH2 is involved in inhibiting the expression of element 1-silencing transcription (REST) factor and Polycomb histone, both of which play important roles in the progression of NEPC (103). There are also reports that the implicit transcriptional activation domain (EZH2TAD) of EZH2 binds to AR and AR splice variant 7 (AR-V7), mediating the assembly and/or recruitment of transcriptional activation related mechanisms at genomic sites lacking PRC2 binding. EZH2 targeted proteolysis targeting chimera (PROTAC) is a potential attractive therapeutic approach for treating invasive prostate cancer that relies on EZH2 and AR connected circuits (106). Drugs targeting EZH2 (Lirametostat, Mevrametostat, Tazemametostat, Valemetostat) are currently being explored in clinical trials for their dosage and combination therapy. In addition, the drug ORIC-944, which directly targets Polycomb histone, has also entered clinical dose exploration.

Lysine specific demethylase 1 (LSD1, also known as KDM1A), a flavin adenine dinucleotide (FAD) -dependent demethylase, is a transcription inhibitory factor that regulates enhancers based on augmented reality technology LSD1 also induces CENPE (a centromere binding protein and mitotic drive protein) through epigenetic programming to promote CRPC (103). A clinical trial with a novel LSD1 inhibitor CC-90011 was already completed and the results are still to be announced.

Histone Deacetylase (HDACs) can remove acetylation of histones. HDAC2 expression is positively correlated with higher Gleason scores of PCa, while the expressions of HDAC1, HDAC2, and HDAC3 are positively associated with the proliferative marker Ki67 (107). HDAC5 loss conferred resistance to CDK4/6 inhibitors such as palbociclib in prostate tumors *in vitro* and *in vivo* by impairing tumor-suppressor protein(RB), but this effect was overcome by the BET-CBP/p300 dual inhibitor NEO2734 (108). The 40 mg Panobinostat (a histone deacetylase inhibitor)/bicalutamide regimen increased rPF survival in CRPC patients resistant to second-line antiandrogen therapy. And epigenetic HDACI therapy reduces AR-mediated resistance to bicalutamide in CRPC models with clinical benefit in patients (109).

Proteins of the bromodomain and extra-terminal (BET) domain family are epigenetic readers that bind acetylated histones through their bromodomains to regulate gene transcription (Selective inhibition of the BD2 bromodomain of BET proteins in prostate cancer). A phase Ib study evaluated the safety and efficacy of GS-5829 alone or in combination with enzalutamide for the treatment of mCRPC, showing good tolerability but limited efficacy, and no significant increase in blood drug concentration was observed (110). ZEN-3694 plus enzalutamide demonstrated acceptable tolerability and potential efficacy in patients with ASI-resistant mCRPC. Further prospective study is warranted including in mCRPC harboring low AR transcriptional activity (111). Its 2b study with enzalutamide is now recruiting. Birabresib has dose-proportional exposure and a favorable safety profile, with clinical activity observed in nuclear protein in testis midline carcinoma (NMC). Further validation is needed in CRPC (112).

Above all, the mainly drugs of targeted therapy in CRPC are summarized in **Table 1**.

**Table 1:** Mainly drugs of targeted therapy in CRPC.

Targeted way	Drug	Target	Phase	NCT identifier	State	Result
Targeting AR signaling pathway	Abiraterone acetate (ABI)	CYP17	3	NCT00268476	Active, not recruiting	OS: 76.6 mo[95% CI 68.7 ~ 86.9]
	enzalutamide + ABI + prednisolone	CYP17, AR	3	NCT00268476	Active, not recruiting	OS: 73.1 mo [95% CI 61.9 ~ 81.3]
	Abiraterone Acetate+ Niclosamide+ Prednisone	CYP17	2	NCT02807805	Active, not recruiting	No Result
	Orteronel (TAK-700)	CYP17	2	NCT01046916	Completed	Kaplan-Meier estimates of freedom from PSA progression: 57% and 42% at 12 and 24 months
	Orteronel (TAK-700)	CYP17	1/2	NCT01666314	Completed	Orteronel 200 mg, tablets, orally, twice daily in 28 day cycles ——PSA50:50%
	galeterone (TOK-001)	CYP17	1	NCT00959959	Completed	No Result
	galeterone (TOK-001)	CYP17	2	NCT02438007	Terminated	No Result
	galeterone (TOK-001)	CYP17	3	NCT01709734	Terminated	No Result
	Enzalutamide (ENZ)	AR	1	NCT03927391	Completed	120 mg OD ENZ owns lower fatigue after 24 wk(difference FACIT-Fatigue 6.2)
	Enzalutamide (ENZ)	AR	2	NCT06015321	Not yet recruiting	No Result
	Apalutamide (APA)	AR	3	NCT01946204	Active, not recruiting	Median OS 73.9 mo
	Apalutamide (APA)	AR	4	NCT04108208	Active, not recruiting	No Result
	Apalutamide + Golimumab	AR, TNF-α	2	NCT05960578	Recruiting	No Result
	Apalutamide + Carotuximab	AR, ENG	2	NCT05534646	Recruiting	No Result
	Darolutamide (DAR)	AR	2b	NCT04157088	terminated	terminated without initiating the randomized comparison
	Bavdegalutamide (ARV-110)	AR	1/2	NCT03888612	Completed	No Result
	ARV-110 and Abiraterone	AR	1b	NCT05177042	Active, not recruiting	No Result
Targeting DNA damage response pathways	Olaparib	PARPs	2	NCT01682772	Completed	400 mg cohort 37% PSA50
	Olaparib	PARPs	4	NCT05457257	Active, not recruiting	No Result
	Olaparib + Abiraterone	PARPs	3	NCT03732820	Active, not recruiting	Median OS: 42.1 mo
	Olaparib + Cediranib	PARPs, VEGF	2	NCT02893917	Active, not recruiting	median rPFS of 10.6 mo
	Rucaparib	PARPs	3	NCT02975934	Completed	rPFS (with or without BRCA alteration)(11.2 mo AND 6.4 mo)
	rucaparib + nivolumab	PARPs + PD-1	2	NCT03338790	Completed	among BRCA1/2-positive populations, ORR: 33.3%
	Talazoparib	PARPs	2	NCT03148795	Completed	objective response rate was 29.8% [95% CI 21.2-39.6]
	Talazoparib + Enzalutamide	PARPs, AR	3	NCT03395197	Active, not recruiting	median rPFS was not reached [95% CI 27.5 mo - not reached]
	Niraparib	PARP1/2	2	NCT02854436	Completed	objective response rate in the measurable BRCA cohort was 34.2% (95% CI 23.7-46.0)
	Niraparib + Abiraterone +Prednisone	PARP1/2	3	NCT04497844	Active, not recruiting	No Result
	Pamiparib	PARP1/2	2	NCT03712930	Terminated	OS: 5.8mo ; rPFS: 2.6 mo; Clinical Benefit Rate:25%
	M3814 + Radium-223	DNA-PKc, bone microenvironment	1/2	NCT04071236	Recruiting	No Result
	CC-115 + ENZ	mTORC1/2 and DNA-PK	1b	NCT02833883	Completed	Median time-to-PSA progression was 14.7 mo; median rPFS was 22.1 mo
Targeting PSMA	177Lu-PSMA-617	PSMA	3	NCT03511664	Completed	rPFS (median, 8.7 vs. 3.4 mo); OS (median, 15.3 vs. 11.3 mo
	177Lu-PSMA-617	PSMA	2	NCT05670106	Active, not recruiting	No Result
	225Ac-J591	PSMA	1	NCT03276572	Completed	safety and preliminary efficacy signals are demonstrated
	177Lu-J591	PSMA	Early 1	NCT04576871	Active, not recruiting	No Result
	177Lu- J591 + Docetaxel/​Prednisone + Fractionated	PSMA	1	NCT00916123	Completed	accurate targeting of known sites of disease and a strong preliminary efficacy signal was observed.
	177Lu-J591 + 177Lu-PSMA-617	PSMA	1/2	NCT03545165	Terminated	PSA decline: 83.3%
	PSMA-ADC	PSMA	2	NCT01695044	Completed	PSA response (30% )29% for Chemotherapy-experienced, 32% for Chemotherapy-naive
Targeting bone microenvironment	Bone modifying agent(BMA)	bone microenvironment	4	NCT04549207	Active, not recruiting	No Result
	Radium-223 dichloride (radium-223)	bone microenvironment	2	NCT05133440	Active, not recruiting	No Result
	zoledronic acid	bone microenvironment	2/3	NCT01006395	Completed	No Result
	zoledronic acid	bone microenvironment	3	NCT00869206	Completed	2 years 1 skeletal-related event: 29.5%(every 4-week), 28.6%(every 12-week)
	Denosumab	RANKL	3	NCT01824342	Completed	better Performance Status
	Denosumab + Enzalutamide	RANKL, AR	2	NCT03869762	Terminated	no Result
Targeting cell cycle	Abemaciclib + Abiraterone	CDK4/6	3	NCT05288166	Active, not recruiting	No Result
	Abemaciclib	CDK4/6	2	NCT04408924	Completed	DCR 45.5%
	Ribociclib + Docetaxel + Prednisone	CDK4/6	1/2	NCT02494921	Completed	rPFS at 6 Months: 65.8% ORR 23.1%
	Ipatasertib + Abiraterone	AKT, AR	3	NCT03072238	Completed	median rPFS: 18.5 mo in the ipatasertib-abiraterone group(patients with PTEN loss)
	Ipatasertib	AKT	1/2	NCT04737109	Terminated	No Result
	Capivasertib + abiraterone acetate	AKT, AR	2	NCT05593497	Recruiting	No Result
	Capivasertib + Docetaxel	AKT	3	NCT05348577	Active, not recruiting	No Result
	Gedatolisib + Darolutamide	pi3k /mTOR	1/2	NCT06190899	Recruiting	No Result
Targeting epigenetic marks	Lirametostat(CPI-1205)	EZH2	1b/2	NCT03480646	Unknown status	No Result
	Mevrometostat(PF-06821497)	EZH2	3	NCT06629779	Recruiting	No Result
	Mevrometostat(PF-06821497)	EZH2	3	NCT06551324	Recruiting	No Result
	Mevrometostat(PF-06821497)	EZH2	1	NCT03460977	Recruiting	No Result
	Tazemetostat(EPZ-6438)	EZH2	1a/1b	NCT04846478	Active, not recruiting	No Result
	Valemetostat (DS-3201)	EZH2	Ib	NCT04388852	Recruiting	No Result
	ORIC-944	PRC2	1	NCT05413421	Recruiting	No Result
	CC-90011	LSD1	1	NCT04628988	Completed	No Result
	Pocenbrodib	p300/CBP	1b/2a	NCT06785636	Recruiting	No Result
	FT-7051		1	NCT04575766	Terminated	No Result
	ZEN003694	BET	1b/2a	NCT02711956	Completed	No Result
	ZEN003694	BET	2b	NCT04986423	Recruiting	No Result
	Birabresib (MK-8628/OTX015)	BET	Ib	NCT02259114	Completed	phase II dose of birabresib in patients with select solid tumors is 80 mg once daily with continuous dosing
	panobinostat	HDCAs	1	NCT00878436	Completed	free of progression and without symptomatic deterioraion:LBH 40mg 42% and LBH 20mg 19% serious adverse event:LBH 40mg 39.29% and LBH 20mg 20.83%
	Entinostat	HDCAs	1	NCT03829930	Terminated	No Result

*OR*, odds ratio; *PSA50*, a decrease in prostate-specific antigen (PSA) of 50% or more from baseline; *mo*, months; *wk*, weeks; *rPFS*, Radiographic Progression-Free Survival; *ORR*, overall response rate; a complete response (CR) and/or partial response (PR) *DCR*, Disease control rates, the percentage of participants with confirmed soft tissue best overall response of CR, PR, or stable disease (SD), and do not have concurrent bone disease progression.

## 3 Immune therapy in CRPC

### 3.1 Immune checkpoint inhibitors

**Figure 2:**
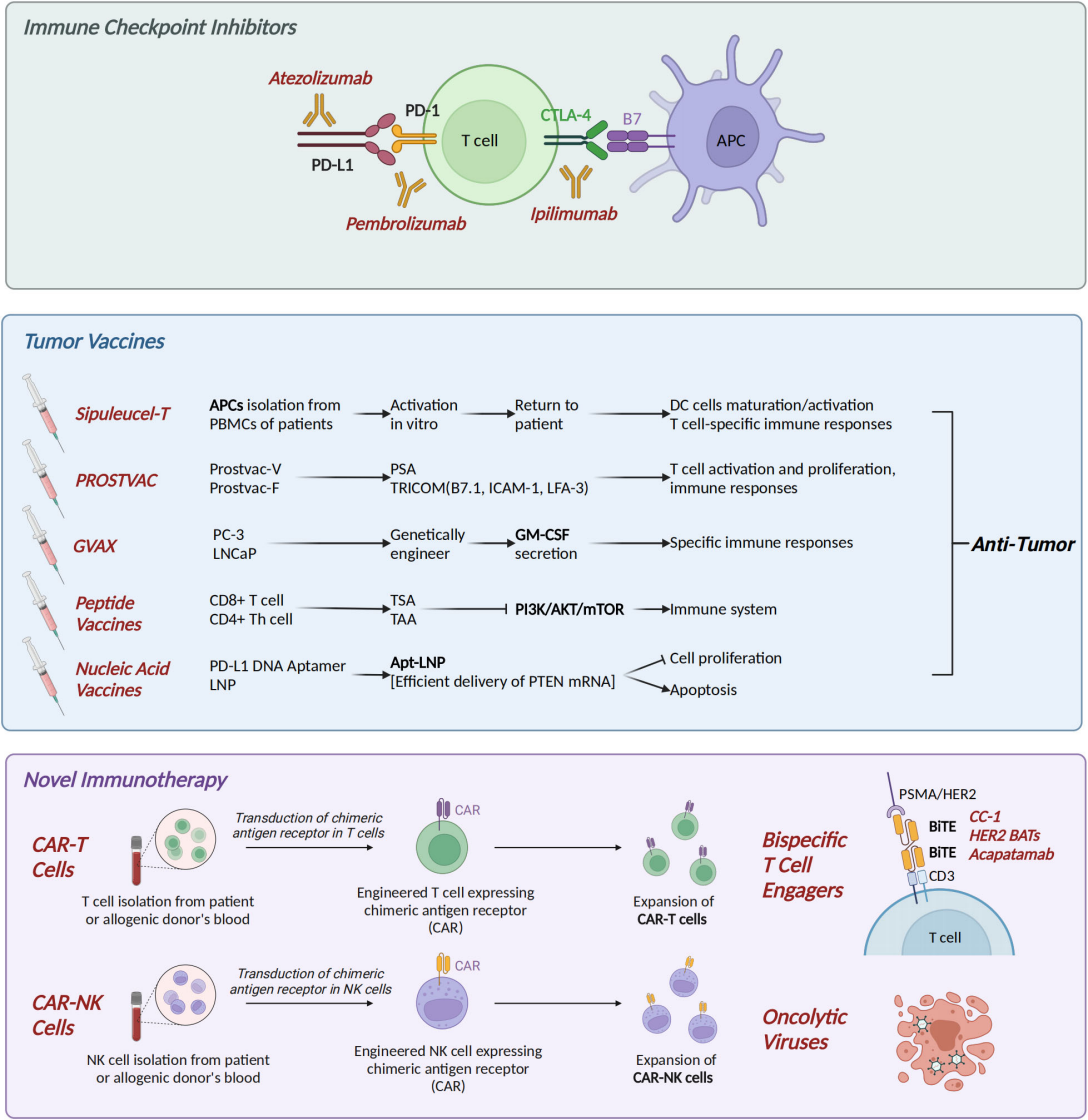
Immune therapy in CRPC. (1) Immune checkpoint inhibitors (ICIs): CTLA-4 inhibitor ipilimumab, PD-1 inhibitor pembrolizumab, and PD-L1 inhibitor atezolizumab. (2) Tumor vaccines: Dendritic cell vaccine Sipuleucel-T; PROSTVAC, consisting of Prostvac-V and Prostvac-F; GVAX (GM-CSF-secreting PC-3/LNCaP cells); peptide vaccines targeting tumor antigens; and Apt-LNP [PTEN mRNA] nucleic acid vaccine. (3) Novel immunotherapies: Chimeric antigen receptor (CAR) T/NK cells, bispecific T-cell engagers (BiTEs), and oncolytic virus therapy. CRPC, castration-resistant prostate cancer. Created in https://BioRender.com.

Immune checkpoints are a class of regulatory signaling molecules in the immune system that maintain immune homeostasis and prevent self-tissue damage caused by excessive immune responses by inhibiting or activating the activity of immune cells. They include cytotoxic T lymphocyte antigen 4 (CTLA-4), programmed cell death protein 1 (PD-1), lymphocyte activation gene-3 (LAG-3), and T cell immunoreceptor with Ig and ITIM domain (TIGIT) (**Figure 2**). For example, immune checkpoint inhibitors (ICIs) block inhibitory signaling pathways such as PD-1/PD-L1 and restore anti-tumor immune activity. In this article, we mainly elaborate on the roles and therapeutic applications of CTLA-4 and PD-1 in CRPC.

**1) Cytotoxic T Lymphocyte Antigen 4**

CTLA-4 is a critical ICIs that plays a pivotal role in regulating T cell activation and maintaining immune homeostasis. Ipilimumab is a human monoclonal antibody (mAb) that functions by inhibiting CTLA-4, thereby deregulating the inhibition of T-cell activation through competitive blocking of the binding of CTLA-4 to B7 ligand (CD80/CD86) on the surface of antigen-presenting cells (APCs) (113). A study of 30 samples found that ipilimumab in CRPC patients with low Tumor Mutational Burden (TMB) could achieve long-term survival benefits through activation of local T-cell immunity, suggesting that immune microenvironment profiling (rather than TMB alone) may be more suitable for screening populations for potential benefit from Immune checkpoint blockade (ICB) (114).

Although ICIs have been shown to significantly prolong patient survival in some solid tumors, their efficacy in prostate cancer has been limited. A potential avenue for enhancing the effectiveness of ICIs in prostate cancer is through combination therapy with other therapeutic modalities, including vaccines, hormone therapies, PARP inhibitors, and chemotherapy. The CheckMate 650 trial (NCT02985957) evaluated the efficacy of nivolumab (1 mg/kg) in combination with ipilimumab (3 mg/kg) in patients with mCRPC pre-chemotherapy (cohort 1, n=45) and post-chemotherapy (cohort 2, n=45), obtaining an ORR of 10% in the post-chemotherapy cohort and OS of 15.2 months (115). A phase III trial of 799 samples was conducted to analyze the efficacy of ipilimumab in combination with radiotherapy. The results demonstrated that ipilimumab in combination with radiotherapy exhibited a significant long-term survival benefit compared to the placebo. This benefit was observed in patients with mCRPC that progressed after docetaxel treatment. Following a median follow-up extension of 2.4 years, the long-term OS rate was found to be significantly higher in the ipilimumab group (25.2%) than in the placebo group (16.6%) (116). As has been documented, the inhibition of STAT3 has been shown to enhance the anti-tumor efficacy of anti-CTLA-4 treatment for prostate cancer. This finding has been corroborated in murine models (117).

**2) Programmed cell death protein 1 and programmed Death-Ligand 1**

The protein known as PD-1, in conjunction with its ligand, PD-L1, functions as a negative regulator of T-cell function, thereby maintaining a balance between T-cell activation, tolerance, and immune-mediated tissue damage. In contrast to CTLA-4, the role of PD-1 does not involve interference with co-stimulation; rather, it interferes with signaling mediated by the antigen receptor of the T cell. In addition, one of its ligands, PD-L1, can be expressed on a wide range of cell types, including T cells, epithelial cells, endothelial cells, and tumor cells after exposure to the cytokine interferon-gamma (IFN-γ) produced by activated T cells (118). Elevated PD-L1 expression in certain tumor cells is associated with immune escape from tumor cells (119).

Pembrolizumab is a groundbreaking ICI that targets the PD-1 receptor. It is widely used in cancer immunotherapy to enhance the immune system’s ability to fight tumors. A combined Meta-analysis of 13 studies with 2533 patients reported that anti-PD-1/PD-L1 combination therapy may significantly increase PFS benefit, however, the overall survival of patients with CRPC warrants further testing (120). Pembrolizumab in KEYNOTE-921 (NCT03834506) in combination with docetaxel and prednisone or prednisolone did not differ in OS in patients with CRPC (121). According to the phase 2 KEYNOTE-199 study (NCT02787005, cohorts 4 and 5), the addition of pembrolizumab to ENZ treatment showed some antitumor activity in patients with CRPC (122). However, in another study (phase 1b/2 KEYNOTE-365, cohort C, n=102), pembrolizumab + ENZ showed limited antitumor activity in patients with CRPC (123). However, in patients with metastatic prostate cancer, ENZ plus atezolizumab failed to prolong survival (124). This suggests the need to develop precise screening criteria based on biomarkers such as PD-L1 expression, tumor mutational load, or T-cell infiltration characteristics to optimize therapeutic decision-making for immune checkpoint inhibitors across the full spectrum of prostate cancer stages.

The FDA has approved the use of pembrolizumab for the treatment of adult and pediatric patients with unresectable or metastatic microsatellite instability-high (MSI-H) cancer (125). In comparison with high tumor mutational burden without microsatellite instability (TMB-H/MSS) prostate cancer, MSI-H/dMMR prostate cancer has been shown to have a higher TMB, indel, and neoantigen burden. These differences may contribute to a deeper and longer-lasting response to treatment with pembrolizumab (126). Another tool, the tumor immune contexture score (TICS), has been shown to refine existing risk stratification systems and provide ideas for ADT and immunotherapy for prostate cancer (127). Sigma uses a multiclass gradient boosting classifier to categorize samples into mismatch repair deficient (MMRd) or mismatch repair proficient (MMRp). MMRd status is associated with significant survival improvement and durable remission, and SigMA-based MMRd assays allow for more comprehensive screening of patients likely to benefit from pembrolizumab treatment.

However, the ineffectiveness of ICIs in patients with desmoplasia-resistant CRPC is partly attributable to the promotion of immune evasion by myeloid-derived suppressor cells (MDSCs) within the tumor microenvironment (128). A study demonstrated that a class of tumor-associated macrophages (SPP1 hi-TAMs), which highly express SPP1 and secrete adenosine to activate the A2AR signaling pathway, emerged in metastatic desmoplasia-resistant prostate cancer, leading to ICIs resistance *in vivo* (129). This finding was corroborated in a phase I clinical trial, which demonstrated that the combination of the A2AR antagonist ciforadenant and atezolizumab exhibited superior efficacy compared to atezolizumab monotherapy in a specific patient subset.

Above all, the mainly ICIs involved in CRPC are summarized in **Table 2**.

**Table 2:** Mainly ICIs involved in CRPC.

Drug	Target	Phase	NCT identifer	Status	Result
Ipilimumab	CTLA-4	2	NCT02113657	Completed	PFS 1.7 months; OS 24.3 months
Ipilimumab	CTLA-4	3	NCT01057810	Completed	PFS 5.59 months; OS 28.65 months
Ipilimumab + Nivolumab	CTLA-4, PD-1	2	NCT02985957	Completed	ORR 10%; OS 15.2 months
Ipilimumab + Radiotherapy	CTLA-4	3	NCT00861614	Completed	PFS 4.01 months; OS 11.04 months
Pembrolizumab + Docetaxel	PD-1, tubulin	3	NCT03834506	Completed	rPFS 8.6 months; OS 19.6 months
Pembrolizumab + Enzalutamide	PD-1, AR	2	NCT02787005	Completed	rPFS 4.2 months; OS 18.9 months
Pembrolizumab + Enzalutamide	PD-1, AR	1b/2	NCT02861573	Recruiting	–

### 3.2 Tumor vaccines

**(1) Sipuleucel-T (Provenge^®^)**

Sipuleucel-T (Sip-T) represents a significant development as the first dendritic cell vaccine to be approved by the FDA for the treatment of advanced prostate cancer (130). Activated antigen-presenting cells (APCs) were prepared by obtaining patient peripheral blood mononuclear cells (PBMCs) and co-culturing them *in vitro* with PA2024, a fusion protein of prostatic acid phosphatase (PAP) and granulocyte-macrophage colony-stimulating factor GM-CSF (131). The PAP in PA2024 is presented as a tumor-associated antigen by the dendritic cells, and their maturation and activation are enhanced by GM-CSF. CSF has been demonstrated to enhance their maturation and activation, in addition to inducing T-cell specific immune responses (132).

An analysis of patient samples obtained from three Sip-T trials revealed that antigen-specific cytotoxic T-lymphocyte (CTL) responses against PA2024 and PAP were significantly enhanced in peripheral blood cells of patients after treatment, which was strongly associated with an improvement in OS (133). Three phase III clinical trials (N=737) demonstrated that Sip-T induced an immune response through the *in vitro* activation of APCs: APC activation (CD54 up-regulation) increased 6.2-fold, 10.6-fold, and 10.5-fold at the first, second, and third administrations, respectively. Antigen-specific T cells were detected after the first administration, and 78.8% of the patients produced an antigen-specific immune response. Both the number of cumulative APC activation levels and antigen-specific immune response were significantly correlated with OS (134). A detailed analysis of patient samples from two clinical trials (NCT01431391 and NCT01981122) was conducted, which revealed that Sip-T induced antigen-specific CD8^+^ T and CD4^+^ T responses against PA2024 and PAP (135). In a double-blind, placebo-controlled, multicentre phase III trial (NCT00065442), 512 patients with mCRPC were randomized 2:1 to receive Sip-T or placebo. The results demonstrated a 22% reduction in the risk of death in the Sip-T group compared to the placebo group, with a median OS prolongation of 4.1 months. There was no difference between the two groups in the time to objective disease progression (130). In two randomized, double-blind, placebo-controlled phase III trials, 225 patients with advanced prostate cancer were treated with Sip-T or placebo in a 147:78 ratio. The risk of death was reduced by 33% in the Sipuleucel-T group. Common adverse events included chills, fever, headache, malaise, dyspnoea, vomiting, and tremor (136).

The survival benefit of Sip-T monotherapy remains limited, but further optimization of the regimen is required to balance efficacy and safety. A phase II clinical trial of 54 patients with CRPC demonstrated that IL-7-amplified lymphocyte populations may enhance the immune response to Sip-Tin patients with metastatic desmoplasia-resistant prostate cancer. The study revealed that 31% of patients in the rhIL-7 group achieved a PSA doubling time of more than six months, in contrast to the 14% observed in the observation group (137). The phase II clinical trial (NCT01804465, n=50) demonstrated that Sip-T in combination with the CTLA-4 inhibitor ipilimumab exhibited limited clinical activity in CRPC and that the timing of administration (immediate vs. delayed) did not affect the antigen-specific immune response (138). This indicates a necessity for further exploration of synergistic mechanisms of immunotherapy in the future to enhance the immunotherapeutic benefit of CRPC.

**(2) PROSTVAC**

PROSTVAC employs a recombinant poxvirus vector as the primary immunotherapy and a recombinant chickenpox virus vector as a booster, utilizing a heterologous initiation booster strategy (139). Both vectors contain transgenes for PSA and TRICOM (a triad of T-cell costimulatory molecules: costimulatory molecules B7.1, leukocyte function-associated antigen-3, and intercellular adhesion molecule-1). The PSA-TRICOM vaccine infects APC and produces APC surface-expressed proteins in the immune environment. The interaction of these APCs with T cells initiates a targeted immune response and T-cell mediated tumor cell destruction (140).

A Phase I trial of recombinant vaccinia prostate-specific antigen (rV-PSA) was conducted on 42 patients with CRPC, in which GM-CSF was combined with the vaccine to enhance the immune response (141). The results demonstrated that the vaccine was well tolerated, with an increase in PSA-specific T cells observed in three of the five patients who were evaluable (142). However, a subsequent phase III trial revealed that PROSTVAC had no impact on either OS or adverse events (AEs) in CRPC (143). Furthermore, biopsies of 10 patients with recurrent prostate cancer treated with the PROSTVAC vaccine demonstrated that the vaccine had a limited and controlled cytotoxic effect on cells expressing natural PSA (144). Furthermore, of the 104 patients who were tested for T-cell response, 57% exhibited a ≥2-fold increase in PSA-specific T cells 4 weeks after vaccination in comparison to their pre-vaccination levels, while 68% demonstrated an immune response to tumor-associated antigens absent from the vaccine (antigen spreading) following vaccination (145). The recombinant poxvirus vaccine exhibited a substantial survival benefit in 32 patients with CRPC, particularly in those with a favorable prognosis (Halabi predicted survival ≥18 months). Alterations in Treg inhibitory function may represent a pivotal immune mechanism for vaccine efficacy, which was not further enhanced by the incorporation of GM-CSF (146).

In the multicentre, randomized clinical trial that was conducted in order to make a comparison between ARA flutamide +/- PROSTVAC as a means of treating CRPC (NCT00450463, n=64), it was demonstrated that flutamide in combination with PROSTVAC therapy did not improve outcomes in patients with CRPC in comparison with flutamide alone (147). Moving forward, there is a clear need to enhance the clinical translational potential of such therapeutic approaches through multidimensional immunomodulation and precise patient stratification.

**(3) GVAX**

The GVAX vaccine is composed of two allogeneic prostate cancer cell lines (PC-3 and LNCaP) that have been genetically modified and irradiated, resulting in the secretion of GM-CSF (148).

Immunotherapy on the GVAX platform was administered to 80 patients with metastatic hormone-refractory prostate cancer using two allogeneic prostate cancer cell lines modified to secrete GM-CSF. The median survival of patients in the high-dose group was significantly better than that of the medium- and low-dose groups; only 1 patient had a PSA reduction >50%, suggesting limited efficacy of a single agent (149).

The GVAX vaccine comprises genetically modified tumor cells that are modified with GM-CSF, which acts at the site of vaccination to enhance dendritic cell activation, as well as antigen presentation to the b- and t-cell arms of the immune system (150). A study was conducted to evaluate the efficacy of GVAX versus docetaxel chemotherapy in CRPC patients. The study concluded that there was no improvement in OS in patients treated with GVAX compared to those treated with docetaxel. Furthermore, the combination of docetaxel chemotherapy with or without GVAX in patients with CRPC revealed that patients in the combination treatment group exhibited a higher mortality rate compared to those receiving docetaxel monotherapy (151). Consequently, both trials were halted, and the clinical development of GVAX for prostate cancer was suspended.

**(4) Peptide vaccines**

Peptide tumor vaccines consist of amino acid sequences of either tumor-specific antigens (TSA) or tumor-associated antigens (TAA). These antigens are designed to activate the immune system, thereby inducing the recognition and elimination of cancer cells. The majority of peptide vaccines target TAA or TSA by stimulating CD8^+^ T cells or CD4^+^ helper T cells through epitope peptides (152). TSA is expressed exclusively in tumor cells; however, TSA recognition is challenging due to tumor and patient heterogeneity. Conversely, TAA is highly expressed in tumor cells and low in normal cells, making it a potential target for cancer vaccines (153).

A Phase I trial (NCT05010200) is currently ongoing to assess the safety and tolerability of a personalized pgv001-based peptide vaccine in combination with CDX-301 in patients with a history of prostate cancer. Another phase I trial (NCT04701021) evaluated the safety, tolerability, immune response, and preliminary clinical outcomes of different doses of the TENDU vaccine in patients with recurrence after primary radical prostatectomy; the results are not yet available. A novel peptide tumor vaccine, KRM-20, in combination with chemotherapy, demonstrated good safety and immunogenicity in CRPC (N=50) but failed to translate into significant clinical benefit (154). Subgroup analyses suggest that specific patient groups (e.g., high lymphocytes, low PSA) may benefit, providing a potential direction for precision immunotherapy.

A randomized, double-blind, placebo-controlled phase 3 trial involving 306 patients treated with personalized peptide vaccination (PPV) in patients with CRPC was conducted. The median OS was not significantly different between the PPV group and the placebo group. However, subgroup analyses indicated that patients exhibiting a low neutrophil percentage or a high lymphocyte percentage at baseline might potentially benefit from PPV treatment (155). A phase II randomized controlled trial (n = 72) was conducted to compare the efficacy of PPV therapy in combination with dexamethasone and dexamethasone alone for the treatment of chemotherapy-naïve CRPC. The results demonstrated that the PFS was significantly longer in the vaccinated group than in the dexamethasone group (22.0 months vs. 7.0 months; p = 0.0076). The median OS was also significantly longer in the vaccinated group (73.9 months vs. 34.9 months; p = 0.00084) (156).

**(5) Nucleic acid vaccines**

Anti-PD-L1 DNA aptamer-coupled lipid nanoparticles (Apt-LNP [PTEN mRNA]) have been shown to possess the capability of targeting phosphatase and tensin homolog (PTEN) mRNA to CRPC cells and precisely regulating the PTEN-PI3K/AKT signaling axis. *In vitro*, assays have demonstrated the significant inhibition of tumor progression that can be achieved through the use of these nanoparticles (157). Preclinical studies have demonstrated that pTVG-AR enhances antigen-specific CD8 T-cell responses and delays prostate cancer progression and the emergence of desmoplasia-resistant disease (158). The combination of nivolumab and the pTVG-HP vaccine (which targets prostatic acid phosphatase, PAP) was found to be safe and immunologically active in patients with stage M0 PC (NCT03600350, n=19). In this study, 21% of patients experienced a greater than 50% reduction in PSA, with a nonsignificant clinical effect (159). However, a randomized controlled phase II clinical trial (NCT01341652, n=99) found that the DNA vaccine pTVG-HP in combination with GM-CSF adjuvant did not meet the primary endpoint in patients with CSPC. The discrepancy between its immune-activating effect and clinical efficacy suggests the need to combine immune checkpoint blockade or other targeting strategies to overcome suppression by the tumor microenvironment (160).

**(6) Other vaccines**

Adenoviruses (Ads) are a class of DNA viruses with linear double-stranded genomes that contain an icosahedral, unenveloped capsid. Adenovirus type 5 (Ad5) vectors are notable for their inability to integrate (i.e., their genomes remain free) and exhibit a very low risk of germ cell line transmission and/or insertional mutagenesis. These non-replicating adenoviral vectors have the capacity to evade or reduce the neutralizing antiviral immune response and can be consistently boosted to maximize the immune response.

Ad5 PSA/MUC-1/brachyury is a multi-antigen (PSA, MUC-1, brachyury) targeted vaccine based on adenovirus type 5 (Ad5) vectors (NCT03481816), which has demonstrated a favorable safety profile, immunogenicity, and preliminary clinical activity in patients with CRPC, but it has limited single-agent efficacy (161).

A multicenter, randomized trial (NCT02293707, n=98) of GX301, a telomerase-based cancer vaccine, demonstrated good safety and immunogenicity in the treatment of CRPC (162). ADXS31–142 is a Listeria monocytogenes-based attenuated immunotherapy targeting PSA. In the KEYNOTE-046 study (N=50), ADXS31–142 in combination with pembrolizumab demonstrated a PFS of 5.4 months and median overall survival (OS) of 33.7 months (163).

Above all, the mainly tumor vaccines involved in CRPC are summarized in **Table 3**.

**Table 3:** Mainly tumor vaccines involved in CRPC.

Drug	Target	Phase	NCT identifer	Status	Result
Sipuleucel-T	PAP	3	NCT00065442	Completed	OS 25.8months
Sipuleucel-T + Leuprolide Acetate	PAP, GnRHR	2	NCT01431391	Completed	IFN-γ ELISPOT (per 300,000 PBMC) 81.0 or 61.1
Sipuleucel-T + Enzalutamide	PAP, AR	2	NCT01981122	Completed	PA2024 Week 52 16.67 or 25.43 10^3 cells/mL
Sipuleucel-T + Ipilimumab	PAP, CTLA-4	2	NCT01804465	Completed	71.4% or 87.5% PAP and/or PA2024 responses
Sipuleucel-T + rhIL-7	PAP, IL-7R	2	NCT01881867	Completed	31% PSA doubling times of >6 months
PROSTVAC	PSA	3	NCT01322490	Completed	OS 33.2 months
PROSTVAC + Flutamide	PSA, AR	2	NCT00450463	Completed	56% PSA responses
pgv001 + CDX-301	TLR3, Flt3L	1	NCT05010200	Active, not recruiting	No results posted
TENDU	PSA	1	NCT04701021	Completed	No results posted
KRM-20 + Docetaxel + Dexamethasone	TAAs, Tubulin, GR	2	–	Completed	PFS 8.9 months; OS 37.7 months
PPV	TAAs	3	UMIN000011308	Completed	PFS 4..2 months; OS 16.1 months
PPV + Dexamethasone	TAAs, GR	2	UMIN-CTR: 000000959	Completed	PFS 22.o months, OS 73.9 months
Ad5 PSA/MUC-1/brachyury	TAAs	1	NCT03481816	Completed	PFS 22 weeks
GX301	Telomerase	2	NCT02293707	Completed	62% 18 months OS; 48% 24 months OS
adx31-142 + Pembrolizumab	PSA, PD-1	1/2	NCT02325557	Completed	PFS 5.4 months; OS 33.7 months

*GnRHR*, Gonadotropin-Releasing Hormone Receptor; *IL-7R*, Interleukin-7 Receptor; *Flt3L*, Fms-related tyrosine kinase 3 ligand; *TLR3*, Toll-like receptor 3; *GR*, Glucocorticoid receptor.

### 3.3 Novel immunotherapy

(1) Chimeric antigen receptor T cells

Chimeric Antigen Receptor T cells (CAR-T cells) therapy involves the genetic engineering of patients’ autologous T cells to construct chimeric antigen receptors (CARs) on their surface, thereby empowering T cells to specifically recognize tumor cell surface antigens and mediate targeted anti-tumour immune responses (164). The primary targets of CAR-T therapy for PCa include PSMA, B7-H3, prostate stem cell antigen (PSCA), and epithelial cell adhesion molecule (EpCAM) (165). A phase I trial (NCT03089203) using PSMA CAR-T cells carrying dominant-negative TGFβ receptor demonstrated that grade ≥2 cytokine release syndrome (CRS) was observed in 5 out of 13 patients, PSA decreased by ≥30% in 4, and after PSA decreased by >98% in 1 patient, death due to enterococcal sepsis was recorded (166). The rationale behind the use of PSMA-directed CAR-T cells, which are enveloped in dominant negative TGF-β receptors following their translocation to prostate tumors, is that they can act as TGF-β. This, in turn, serves to reduce the impact of an important immunosuppressive component in the local environment and to promote CAR-mediated tumor lysis (167). In a phase I trial that used targeted PSCA to target BPX-601 CAR-T cells, 56% of nine CRPC patients achieved a ≥50% reduction in prostate-specific antigen. However, two dose-limiting toxicities and two treatment-related deaths occurred in the maximum-dose CRPC cohort (168). The initial phase 1 clinical trial of PSCA-targeted CAR-T cells in patients with CRPC (NCT03873805) revealed that a reduced lymphocyte depletion regimen exhibited a favorable safety profile and demonstrated preliminary antitumor activity (169).

The presence of certain structures within solid tumors (e.g., extracellular matrix, tumor stroma) has been demonstrated to impede the contact between CAR-T cells and the tumor itself, thereby reducing the infiltration capacity of CAR-T cells into tumor tissues and their targeted killing efficiency (170). In recent years, the new generation of CAR-T therapies has demonstrated superior tumor clearance efficacy by integrating co-stimulatory molecules to improve the expansion capacity and killing activity of T cells. A study was conducted in which a set of IL23mAb-PSMA-CAR was designed, and it was demonstrated in mice that IL-23 monoclonal antibody (IL-23mAb) combined with PSMA CAR was superior to PSMA CAR alone in eradicating prostate cancer (171).

**(2) Bispecific T cell engagers**

Bispecific T cell articulators (BiTEs), which are engineered bispecific antibodies, represent a novel approach to CRPC immunotherapy. These molecules facilitate the connection between prostate tumors and T cells, thereby directly stimulating the activity of cytotoxic T cells. This process occurs independently of the interaction between the T-cell receptor and the major histocompatibility complex (MHC), thus promoting an immune response against cancer cells (172). These can be designed to target tumor-associated antigens, thereby minimizing damage to healthy tissues and reducing off-target effects (173).CRPC is enriched with our-associated antigens, including, but not limited to, PSMA, PSCA, hK2, and STEAP1 (174).

CC-1 is an IgG-derived PSMA/CD3 BiTE for improved dual targeting of tumor cells and vascular cells in PSMA-positive tumors (175). A phase I clinical trial (NCT04104607) is currently underway to evaluate the safety, tolerability, and preliminary efficacy of CC-1 in patients with desmoplasia-resistant prostate cancer. To overcome the immunosuppressive microenvironment and physical barriers specific to prostate cancer, and to avoid possible adverse effects, multi-targeted approaches will be needed in the future to consider the heterogeneity of tumor antigens (176). HER2 BATs represent a class of targeted HER2 tumor antigens, comprising both anti-CD3 and anti-Her2 bi-armed antibodies, and have been shown to elicit substantial immune responses against cancer cells (177). A phase 2 trial combining HER2 BATs with the immunotherapy agent, pembrolizumab, for the treatment of CRPC, enrolled 14 patients, with a primary endpoint of 6-month PFS rate of 38.5% (178). Acapatamab, a PSMA x CD3 bispecific T-cell armature, was shown to have a significant impact on the immune response to CRPC in a 133-patient phase I clinical trial, showing some anti-tumor activity and suggesting it as a potential therapeutic option for patients with CRPC. However, further validation of its survival benefit and long-term safety is required (179).

**(3) Oncolytic virus therapy**

The capacity of Oncolytic virus (OVs) to lyse tumor cells without affecting normal cells is well documented. However, upon infection of normal cells, viral components trigger an antiviral immune response through multiple mechanisms, leading to the release of soluble antigens, danger signals, and type I interferons, which in turn trigger an antiviral immune response (180). Viruses that have been identified as having an oncolytic effect include adenovirus (Ads), herpes simplex virus (HSV), cowpox virus, vesicular stomatitis virus (VSV), respiratory enteric orphan virus (EWV), Newcastle disease virus (NDV), coxsackievirus, measles virus (MeV), and Sendai virus (Japanese haemagglutinin virus) (181).

The oncolytic alphavirus SFV-VA7 has been identified as a highly promising therapeutic option, with a single intraperitoneal administration of SFV-VA7 achieving a 100% cure rate in subcutaneous and *in situ* LNCaP tumor models (182). In a preclinical study, the lysogenic reovirus strain mutant jin-3 exhibited tumor tropism in a multi-individual prostate cancer model and induced potent lysogenic and immunomodulatory responses, making it an attractive candidate (183). ZD55-SATB1 is a lysogenic adenovirus that targets SATB1, and it was demonstrated that ZD55-SATB1, in combination with Docetaxel, inhibited the proliferation, migration, and invasion of DU145 and PC-3 cells and promoted apoptosis of DU145 and PC-3 cells more than a single agent. In animal models, the combination of ZD55-SATB1, Docetaxel, and endocrine therapy effectively inhibited the growth of transplanted tumors in nude mice, accompanied by elevated expression of caspase-3 and caspase-8, and decreased expression of Bcl-2 and angiogenic marker CD31, compared with other treatment groups (184).

A phase I study (NCT02043665) evaluated the combination of intravenous V937 and pembrolizumab in treating patients with advanced solid tumors. The study demonstrated that the intratumoural administration of lysovirus V937 exhibited both activity and safety. However, the objective remission rate associated with the intravenous administration of V937 pembrolizumab in the extension cohort was not higher than the objective remission rate observed in previous studies with pembrolizumab monotherapy (185).

**(4) Chimeric antigen receptor NK cells**

NK cells have been identified as pivotal mediators of antibody-dependent cell-mediated cytotoxicity (ADCC), as they are able to recognize the IgG Fc fraction bound to tumor cells and subsequently induce their apoptosis by expressing CD16 (186). Chimeric Antigen Receptor NK cells (CAR-NK cells) leverage the innate killing mechanism of NK cells, thereby circumventing the occurrence of graft-versus-host disease (GVHD), and can be prepared using “off-the-shelf” cells (187). However, it should be noted that NK cells have a short half-life (< 10 days), which also means that repeated administration may be required to achieve a durable response (188). In a manner analogous to that of CAR-T cells, PSMA is a highly promising target for CAR-NK cells.

The CD244-based recombinant lentiviral vector constructed with p-PSMA-CAR-NK92MI cells selectively and successfully killed PSMA + target cells and was highly effective against PSMA-positive C4–2 cells and PSMA-negative PC3 cells with specific lysis rates of 73.19% and 33.04%, respectively. The efficacy of this approach was further demonstrated in both *in vivo* and *in vitro* settings, highlighting its potential for addressing CRPC cells (189). In an *in vitro* trial combining treatment with CAR-NK-92 and anti-PD-L1 monoclonal antibody, atezolizumab enhanced the anti-tumor effect of CAR NK-92 by directly acting on PD-L1 on CAR NK-92 and by blocking PD-L1/PD-1 axis, releasing CD8+ T cells, effectively improving the anti-CRPC efficacy (190).

The majority of vaccine therapies have demonstrated favourable safety and biological activity in prostate cancer, yet their clinical activity when employed as a monotherapy is restricted. Consequently, future research should concentrate on combining immunotherapy with other therapeutic modalities to enhance overall efficacy and reduce adverse effects. The development of novel vaccines, such as personalized vaccines based on neoantigens, and the optimization of drug delivery systems, including nanoparticles, represent significant avenues for enhancing efficacy (191).

Despite the demonstrated efficacy of immunotherapy in prostate cancer, its application remains encumbered by significant challenges. Firstly, the occurrence of immune-related adverse events (irAEs), including CRS, a systemic inflammatory response due to over-activation of the immune system, has been observed, with the potential to result in multi-organ failure in severe cases. For instance, CAR-T cell therapy, while specifically targeting PSMA, may lead to normal tissue damage as PSMA is also expressed at low levels in normal tissues. Secondly, although immunotherapy, either as a monotherapy or in combination with other therapies (e.g., chemotherapy, targeted therapies), has been shown to improve the OS of patients, no single combination therapy has been demonstrated to be universally effective for all prostate cancer subtypes. The presence of tumor heterogeneity and the complexity of the immunosuppressive microenvironment represent significant barriers that limit the widespread use and efficacy of immunotherapy.

In summary, while immunotherapy has demonstrated some progress in enhancing patient survival, numerous challenges persist, including the immunosuppressive microenvironment, tumor heterogeneity, and treatment-related toxicity. The development of combination therapy strategies, personalised treatment, and novel immunotherapies is expected to overcome these obstacles and bring more significant clinical benefits to prostate cancer patients. Overall survival (OS) of 33.7 months (163).

Above all, the mainly novel immunotherapy involved in CRPC are summarized in **Table 4**.

**Table 4:** Mainly novel immunotherapy involved in CRPC.

Drug	Target	Phase	NCT identifer	Status	Result
CART-PSMA-TGFβRDN Cells	PSMA, TGFβ	1	NCT03089203	Active, not recruiting	PFS 4.4 months, OS 15.9 months
(PSCA)-targeted CAR-T cells	PSCA	1	NCT02744287	Suspended	Two dose-limiting toxicities and two deaths
(PSCA)-targeted CAR-T cells	PSCA	1	NCT03873805	Active, not recruiting	PSA declines (>30%) in 4 of 14
PSMA/CD3 BiTE (CC-1)	PSMA, CD3	1	NCT04104607	Recruiting	–
HER2 BATs + Pembrolizumab	CD3, HER2, PD-1	2	NCT03406858	Completed	Median PFS 5 months; median survival 31.6 months
Acapatamab (PSMA) x CD3 BiTEs	PSMA, CD3	1	NCT03792841	Completed	PFS 3.3 months
Oncolytic virus (V937)	ICAM-1, DAF	1	NCT02043665	Completed	ORR 6%

*ICAM-1*, intracellular adhesion molecule 1; *DAF*, decay-accelerating factor.

## 4 Discussion

When CRPC progresses to the advanced stage (mCRPC), targeted therapies and immunotherapies still face numerous challenges. Drug resistance driven by complex molecular mechanisms constitutes a major barrier to sustained clinical benefit (4). Among these, the emergence and enrichment of AR-Vs represent the primary cause of resistance to novel endocrine therapies. Degraders, exemplified by PROTACs, no longer rely solely on blocking AR function but instead directly eliminate target proteins, offering a novel approach to overcoming the challenge of AR-Vs. Emerging agents like BMS-986365, a heterobifunctional AR degrader-antagonist, exhibit encouraging activity in heavily pretreated mCRPC patients, underscoring the potential of deeper AR suppression (192). In addition, clinical observations suggest that coagulation factors have a direct role in tumorigenesis and prostate cancer progression (193, 194). Targeting coagulation factor Xa (FXa) synergizes with enzalutamide in preclinical models, suggesting a role for anticoagulants in overcoming resistance (195).

However, current CRPC efficacy remains largely confined to patient subgroups expressing specific biomarkers. Although MSI-H/dMMR or high TMB are clear predictors of ICI efficacy, their prevalence in CRPC is extremely low, leaving the vast majority of patients unable to benefit. Personalized treatment strategies based on distinct patient biomarkers will represent a key future direction.

Immune suppression in the tumor microenvironment and insufficient T-cell infiltration present another major challenge. Prostate cancer typically exhibits low tumor mutational burden (TMB), low lymphocyte infiltration, and abundant immune-suppressive cell populations. This makes it difficult for cytotoxic T cells to be activated, infiltrate, and effectively kill tumor cells. For instance, Sipuleucel-T aims to “prime” the immune response, representing a paradigm shift in treatment; whereas next-generation CAR-T cells incorporate strategies to overcome immunosuppression (such as dominant-negative TGFβ receptors) to maintain function in environments with inhibitory factors. Nevertheless, immune-related adverse events (irAEs) and significant tumor heterogeneity remain prevalent challenges in the clinical application of many immunotherapies.

Despite robust clinical evidence demonstrating survival benefits with triplet (ADT + ARSI + docetaxel) or doublet (ADT + ARSI) therapies in mCSPC (196), real-world adoption remains suboptimal. Current studies indicate only 9.3%-38% of eligible patients receive guideline-recommended combinations, with pronounced disparities: medical oncologists prescribe combinations in 67-88% of cases versus 24-33% by urologists, and Southern U.S. regions show significantly lower uptake than Northeastern/Midwestern areas (197). Systemic barriers—including reimbursement complexity, high drug costs, and limited genomic testing access—contribute to this gap, compounded by clinical misconceptions such as deliberately reserving ARSIs for later-stage disease due to concerns over therapeutic exhaustion. Critically, reduced drug accessibility in early-stage prostate cancer remains a pivotal clinical challenge, demanding urgent healthcare system reforms to bridge evidence-practice gaps.

Then, current clinical decision-making faces complexities due to insufficient comparative data among newer therapies, underscoring the urgency to establish predictive biomarkers for optimizing drug sequencing and minimizing ineffective treatments. Integrating comprehensive molecular profiling into routine practice is essential for advancing precision oncology in CRPC management (198). Unlike breast cancer where routine ER/HER2-based classification enables precision therapy (199), CRPC still lacks clinically actionable molecular subtyping. Although studies identify distinct PAM50-based subtypes such as AR-driven Luminal-A with sensitivity to AR-directed agents and chemotherapy-sensitive Basal-like tumors (200, 201), standardized biomarker algorithms remain unimplemented. Critically, molecular subtyping directly dictates therapeutic efficacy. Luminal B tumors exhibit profound sensitivity to androgen deprivation therapy (ADT), with ADT reducing metastasis risk by 40% (*P*=0.006), while non-Luminal B subtypes show resistance and may even experience harm from ADT (metastasis rate increased from 21% to 37%) (200). The result underscores the urgent need for standardized molecular classification to guide precision interventions and avoid therapeutic mismatches. Lyu et al. identified SPP1hi tumor-associated macrophages as mediators of immunotherapy resistance through adenosine-dependent immunosuppression during prostate cancer progression, proposing SPP1 transcript levels as a potential stratification biomarker (129). Additionally, van Wilpe et al. conducted a phase II trial evaluating nivolumab-ipilimumab in molecularly-selected metastatic CRPC patients. While the overall 6-month disease control rate reached 38%, remarkable efficacy occurred in mismatch repair-deficient (dMMR) patients, contrasting with limited benefits in BRCA-mutated and CDK12-altered subgroups (202).

Furthermore, bioinformatic approaches further contribute to therapeutic discovery. Pan et al. identified circBNC2 as a tumor-suppressive circular RNA significantly downregulated in prostate malignancies. Functional validation confirmed its inhibitory effects on cancer proliferation and migration across experimental models (203). Such findings highlight the dual role of bioinformatics in both biomarker identification and mechanistic exploration.

Collectively, these advancements emphasize the necessity for biomarker-guided therapeutic strategies and multidisciplinary approaches in CRPC management. Future research efforts are expected to yield novel targets and optimized immunotherapeutic combinations through continued molecular characterization and clinical validation.

## 5 Conclusions

Therapeutic advancements in CRPC have transformed its management, with targeted therapies and immunotherapies offering new hope. Second-generation AR inhibitors, PARP inhibitors, and PSMA-targeted agents significantly improve survival, while immunotherapies like checkpoint inhibitors and vaccines rekindle anti-tumor immunity. However, resistance mechanisms-driven by AR splice variants, tumor plasticity, and immune evasion-remains a critical barrier, particularly in mCRPC, underscore the need for combination regimens and biomarker-guided approaches. Emerging strategies, including PROTAC-based AR degraders, epigenetic modulators, and dual-targeting bispecific antibodies, highlight the potential for precision medicine. Moving forward, multidisciplinary strategies combining biomarker-guided therapies, novel immunotherapeutic combinations and molecularly targeted agents are pivotal. In addition, future efforts must prioritize clinical validation of emerging targets, optimization of treatment sequencing, and deeper exploration of tumor microenvironment interactions to achieve durable responses and improve outcomes in CRPC.
